# HCV Subtype Characterization among Injection Drug Users: Implication for a Crucial Role of Zhenjiang in HCV Transmission in China

**DOI:** 10.1371/journal.pone.0016817

**Published:** 2011-02-03

**Authors:** Chiyu Zhang, Nana Wu, Jun Liu, Qinjuan Ge, Yan Huang, Qian Ren, Qingchuan Feng, Guangli He

**Affiliations:** 1 Institute of Life Sciences, Jiangsu University, Zhenjiang, Jiangsu, China; 2 Zhenjiang Center of Disease Control and Prevention, Zhenjiang, Jiangsu, China; 3 Department of Cell Biology and Medicine Genetics, College of Basic Medicine, Zhengzhou University, Zhengzhou, Henan, China; Massachusetts General Hospital, United States of America

## Abstract

**Background:**

HCV transmission is closely associated with drug-trafficking routes in China. However, the transmission route of HCV in Eastern China remains unclear. Here, we investigate the role of Zhenjiang city of Jiangsu province, an important transportation hub linking Shanghai with other regions of China, in HCV transmission.

**Methodology/Principal Findings:**

A total of 141 whole blood samples were collected from injection drug users (IDUs) in Zhenjiang and then tested for HCV infection. Of them, 115 HCV positive plasmas were subjected to RNA extraction, RT-PCR amplification, and sequencing. The subtype characterization and the evolutionary origin of HCV strains circulating in Zhenjiang were determined using polygenetic or phylogeographic analyses. Seven HCV subtypes 1b, 2a, 3a, 3b, 6a, 6e and 6n were detected among Zhenjiang IDUs, showing a complex HCV epidemic. The most predominant subtypes were 3a (38%) and 1b (26.8%). Among these subtypes, subtypes 3b, 6n and 6e originated from Southwestern China (i.e., Yunnan and/or Guangxi), subtypes 2a and 6a from Southern China (i.e., Guangdong), subtype 1b from Central (i.e., Henan) and Northwestern (i.e., Xinjiang) China, and subtype 3a from Southwestern (i.e., Yunnan) and Northwestern (i.e., Xinjiang) China. From Zhenjiang, subtypes 1b and 2a were further spread to Eastern (i.e., Shanghai) and Northern (i.e., Beijing) China, respectively.

**Conclusions/Significance:**

The mixing of seven HCV subtypes in Zhenjiang from all quarters of China indicates that as an important middle station, Zhenjiang plays a crucial role in HCV transmission, just as it is important in population migration between other regions of China and Eastern China.

## Introduction

Hepatitis C virus (HCV) is an enveloped virus and contains a single-stranded positive RNA genome of approximately 9.6 kb in length. HCV belongs to the Hepacivirus genus of the Flaviviridae family [1] and is a main cause for chronic liver disease [2]. HCV infection is a serious global public health problem, with approximately 200 million people estimated to be living with HCV worldwide [3], [4]. In China, approximately 38 million peoples, with a prevalence of around 3.2% of China's population, are estimated to be infected with HCV (http://www.cfhpc.org/detail.asp? id = 283). HCV is transmitted predominantly via injection drug use (IDU), blood transfusion, sexual contact, etc. [3], [5]. Since 1990s when China started to carry out the strict management of blood products and mandatory screening for various blood viruses, HCV infection through contaminated blood products decreased dramatically [6]. Recently, high proportions (15.6–98.7%) of injection drug users (IDUs) were reported to be HCV positive [7], [8], [9], [10], [11], [12]. This indicates that IDU has become the predominant mode of HCV transmission in China [13], [14].

HCV has been classified into six genotypes and a large number of subtypes based on their genomic sequences [15], [16], [17]. Although HCV genotypes 1, 2 and 3 are globally prevalent, the prevalence of other genotypes appears to be segregation of subtypes between different geographic groups [2], [3], [14]. For example, HCV genotype 4 is predominant in Middle East and Northern Africa, and genotype 5 in Southern Africa. For genotype 6, it is geographically predominant HCV genotype in Southeastern Asia [2], [3], [14].

In China, at least 4 genotypes (1, 2, 3, and 6) are found, and the major prevalent subtype is 1b that accounts for about 73% of all HCV infection. Subtype 2a is the second prevalent subtype, accounting for 14%. Genotypes 3 and 6 are geographically predominant HCV genotypes circulating in Southwestern China (e.g. Yunnan) [2], [8]. In addition, subtypes 1a, 1b, 3a, 3b, 6a, 6n, and 6u are circulating among IDUs, with the most predominant subtype of 6a [8], [14].

Zhenjiang, a city of Jiangsu province, is located in the Yangtze River Delta and is one of the most important transportation stations linking Shanghai with other regions of China. Currently, approximately 2214 to 5535 IDUs are estimated to live in Zhenjiang [18]. Previous studies have shown that several HIV-1 subtypes/CRFs are transmitted from Yunnan, a southwestern province, to other regions of China via drug-trafficking routes, in which Jiangsu serves an important station for the spread of HIV-1 to Northern China (Reviewed in Ref. [13]). The investigation of HCV subtype characterization among IDUs in Zhenjiang will help to understand the spreading pattern of HCV infection in Eastern China. Here, we report the HCV genotype distribution among IDUs in Zhenjiang and shed light into the phylogenetic relationship of HCV between Zhenjiang and other regions of China.

## Results

### The amplification of HCV NS5B and C/E2 genomic fragments

Among 141 IDUs who are local residents in Zhenjiang, 115 appear to be HCV positive. The HCV prevalence among this IDUs cohort is 82% (115/141, 95% confidence interval (CI), 88.3%–75.7%), close to the positive ratio (75.1%, 232/309) reported in a recent epidemiological investigation on Zhenjiang IDUs [18]. From 115 positive samples, 65 (56.5%) NS5B fragments (about 1042 bp) and 37 (32.2%) C/E2 fragments (about 1241 bp) were successfully amplified and sequenced. As a result, there are 31 strains containing both NS5B and C/E2 sequences, and 40 strains containing one of both sequences. The failure in the amplification of viral genome in other samples may be due to two days exposure of these samples at room temperature before store at −80°C or low viral loads.

### Prevalent HCV subtypes among IDUs in Zhenjiang, Jiangsu, China

**Figure 1:**
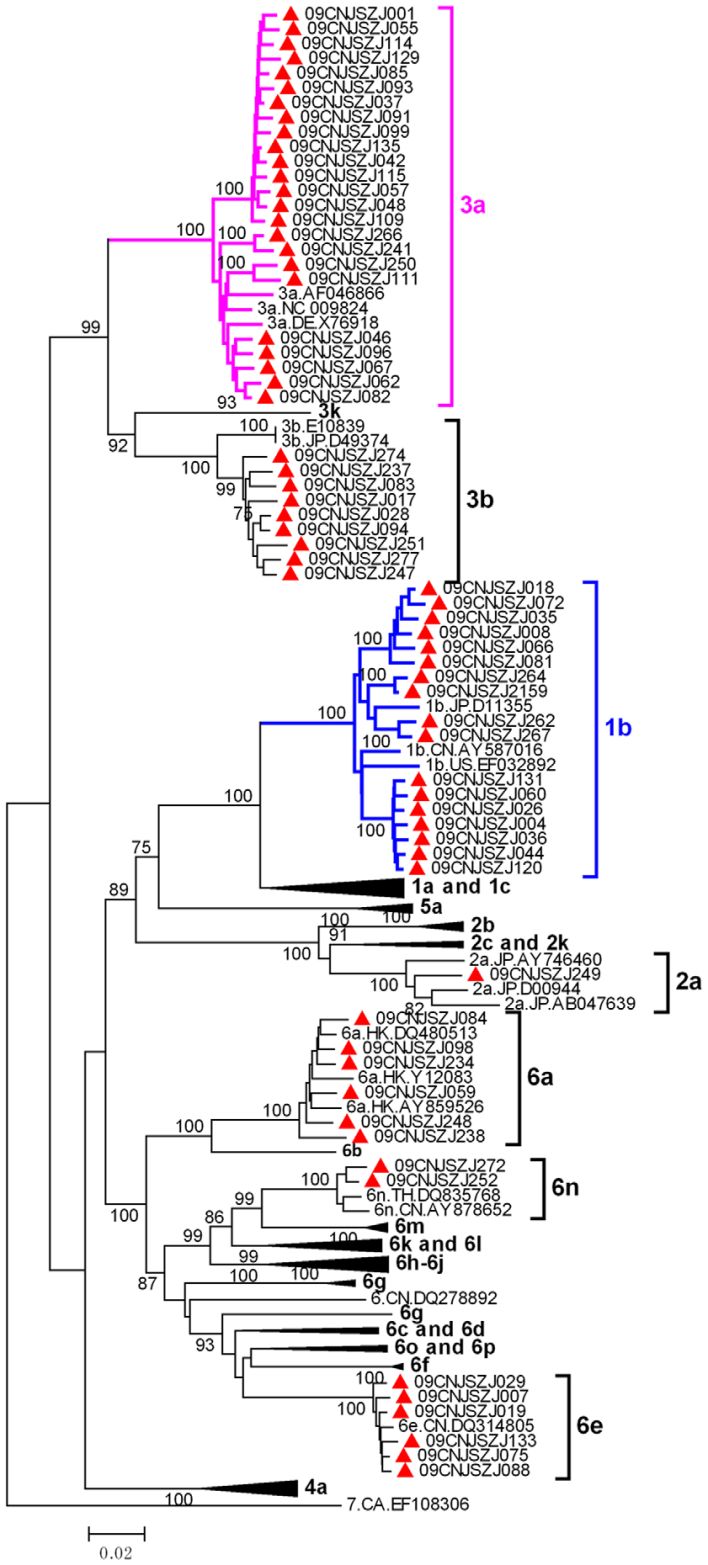
Phylogenetic tree of NS5B fragments of HCV strains isolated from Zhenjiang. The sequences correspond to nucleotides 8262–9312 in HCV H77 genome (NC-004102). Only bootstrap values >75% are shown. The sequences from Zhenjiang IDUs are labeled by red solid triangles.

The phylogenetic trees of 65 NS5B and 37 C/E2 fragments are shown in Figures 1 and S1, respectively. According to the NS5B tree, 7 HCV subtypes including 1b, 2a, 3a, 3b, 6a, 6e and 6n are identified among Zhenjiang IDUs. Except subtype 2a, all subtypes identified in NS5B tree are confirmed in C/E2 tree (Fig. S1). The subtype proportions are well consistent between NS5B and C/E2 fragments (Table 1). The most predominant subtypes among these IDUs are 3a and 1b, both of which account for 38.0% (95% CI, 49.3%–26.7%) and 26.8% (95% CI, 37.1%–16.5%) of all 71 strains (Table 1).

**Table 1:** The distribution of genotypes of Hepatitis C Virus based on NS5B and C/E2 segments.

Genomic fragment	HCV subtypes							Total(n)
	1b	2a	3a	3b	6a	6e	6n	
**NS5B**	17(26.2%)	1(1.5%)	24(36.9%)	9(13.8%)	6(9.2%)	6(9.2%)	2(3.1%)	65
**C/E2**	14(37.8%)	0(0%)	14(37.8%)	3(8.1%)	1(2.7%)	4(10.8%)	1(2.7%)	37
**Total** a	19 (26.8%)(37.1%–16.5%)	1(1.4%)(8%–0%)	27(38.0%)(49.3%–26.7%)	10(14.1%)(22.2%–6.0%)	6(8.5%)(15.0%–2.0%)	6(8.5%)(15.0%–2.0%)	2(2.8%)(10%–0%)	71

a The 95% confidence interval of the distribution ratio (*P*) of each genotype is shown at the bottom of the blank. The interval estimation was performed using normal theory method based on normal distribution (n*P*(1-*P*) ≥5, i.e. subtype 1b, 3a, 3b, 6a and 6e) and exact method based on binomial distribution (n*P*(1-*P*)<5, i.e. subtype 2a and 6n).

### Comparison of HCV subtype distributions among IDUs between Zhenjiang and other regions of China

**Figure 2:**
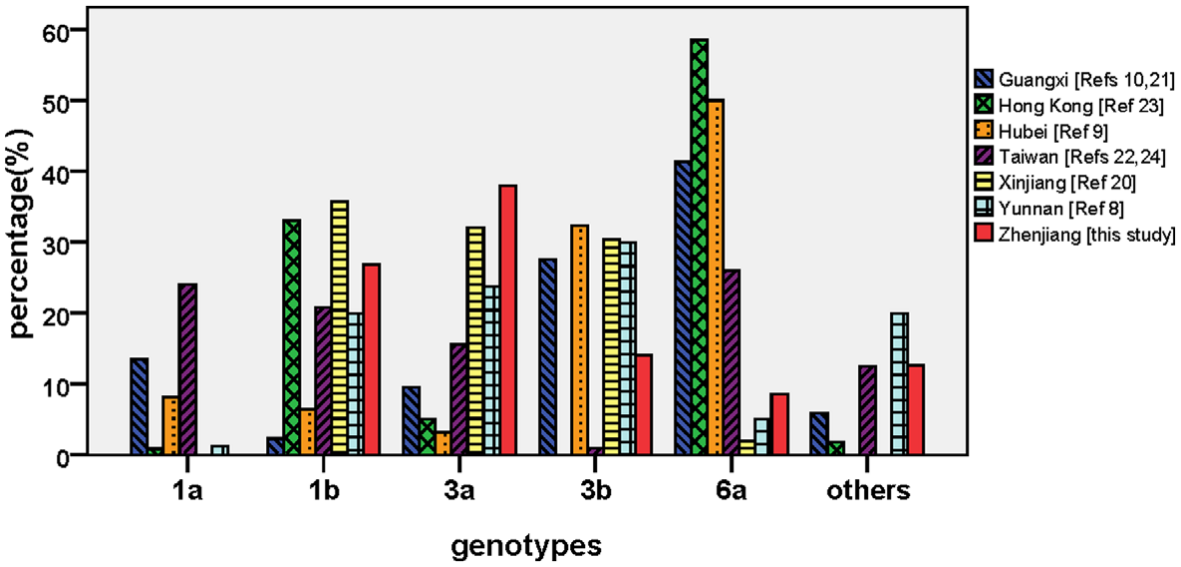
Comparison of HCV subtype distributions among IDUs between Zhenjiang and other regions of China.

HCV transmission among IDUs is associated with various drug-trafficking routes [8], [9], [10], [14], [19]. To investigate whether there is a potential association in HCV transmission among IDUs between Zhenjiang and other regions of China, we collected the distribution information of HCV genotypes from several available provinces of China by the public reference database (PubMed) [8], [9], [10], [20], [21], [22], [23], [24] and compared them with the distribution of HCV subtypes in Zhenjiang. Because of the information unavailable on PubMed, several surrounding provinces such as Anhui, Zhejiang, Shandong and Shanghai are not included in the comparison. Figure 2 shows the HCV subtype distributions among IDUs in Zhenjiang of Jiangsu province and other provinces/regions (including Yunnan, Guangxi, Hubei, Xinjiang, Hong Kong, and Taiwan). One peak in HCV subtype distributions is observed to associate with various provinces/regions. It appears in subtype 6a that is the most predominant HCV subtype circulating in Hong Kong, Hubei and Guangxi. In Zhenjiang, three top predominant subtypes are 3a, 1b and 3b. The three top predominant subtypes observed in Zhenjiang are also the most predominant subtypes in Xinjiang and Yunnan (Fig. 2). Although the patterns of HCV subtype distributions are different between the three regions, the consistence in three top predominant subtypes suggests a potential association of Zhenjiang with Xinjiang and Yunnan in the transmission of at least subtypes 1b, 3a, and 3b (Fig. 2).

### HCV subtypes circulating among Zhenjiang IDUs had complex phylogenetic origins

**Figure 3:**
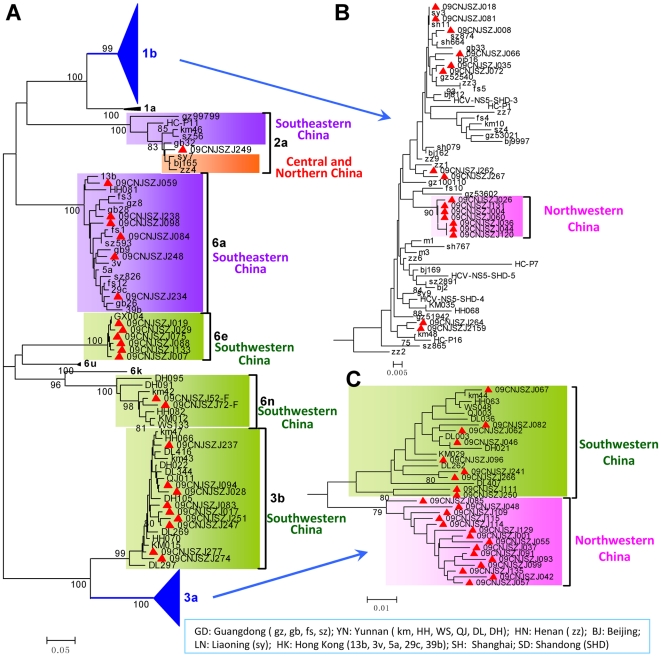
The NS5B phylogenetic relationship of HCV strains isolated from Zhenjiang IDUs with those from other regions of China. Please see Figure 6 for the location of other regions in China and Figure 1 for other details. (A) The whole phylogenetic tree. (B) The sub-tree of HCV subtype 1b strains. (C) The sub-tree of HCV subtype 3a strains.

To investigate the evolutionary origin of HCV subtypes circulating in Zhenjiang, the phylogenetic trees of NS5B (Fig. 3A) and C/E2 fragments (Fig. S2A) were constructed with the sequences from Zhenjiang and other regions of China. Because of few corresponding sequences available from Database, no Taiwan sequence was included in the phylogenetic analyses. In both trees, all subtypes form well-supported clades (with bootstrap values of ≥99%). In subtype 6a clade, all Zhenjiang strains are dispersed among the strains from two southeastern provinces/regions of China, Guangdong and Hong Kong, indicating that 6a subtype circulating among Zhenjiang IDUs might originate from Southeastern China. In the clades of 3b and 6n, all Zhenjiang strains closely cluster within the strains from Yunnan, indicating that 3b and 6n subtypes are introduced into Zhenjiang from Southwestern China. In addition, 6e subtype strains from Zhenjiang cluster with one sequence from Guangxi, implying an evolutionary association in subtype 6e between Zhenjiang and Southwestern China. Intriguingly, in the 2a clade of NS5B tree, one sequence from Guangdong is placed at the root, and one Zhenjiang sequence (09CNJSZJ249) cluster between the strains from Southern China (i.e. Guangdong) and from Central and Northeastern China (Fig. 3A). This implies that Zhenjiang might be a middle station for HCV subtype 2a transmission from Southern China to Central and Northeastern China.

Different from the clades of 2a, 3b, 6n and 6e subtypes, the 1b and 3a clades contain two or more than two sub-clusters. In the 1b clade, the sequences from Zhenjiang are dispersed together with strains from Northeastern, Northern, Central, Eastern, Southeastern and Southwestern China, supporting the nationwide prevalence of subtype 1b. Because of lack of NS5B sequences from northwestern province Xinjiang, we further constructed a 1b sub-tree using the C/E2 sequences from whole China (Fig. S2B). Consistent with the NS5B tree, strains isolated in Zhenjiang are also dispersed together with the strains from whole China in C/E2 tree. In particular, one well-supported sub-cluster shows a close cluster of Zhenjiang HCV strains with the strains from Xinjiang (Fig. S2B). These results suggest that Zhenjiang might be one of the most important mixing places of HCV 1b strains from other regions of China.

With respect to the 3a subtypes, both NS5B and C/E2 trees show that the strains from Zhenjiang are dispersed into two different sub-clusters. One sub-cluster includes HCV strains from Yunnan and another includes strains from Xinjiang (Figs. 3C and S2C). This suggests that 3a strains circulating in Zhenjiang are phylogenetically related to Yunnan and Xinjiang strains (Figs. 3C and S2C).

### Migration routes of HCV 1b and 3a in China

**Figure 4:**
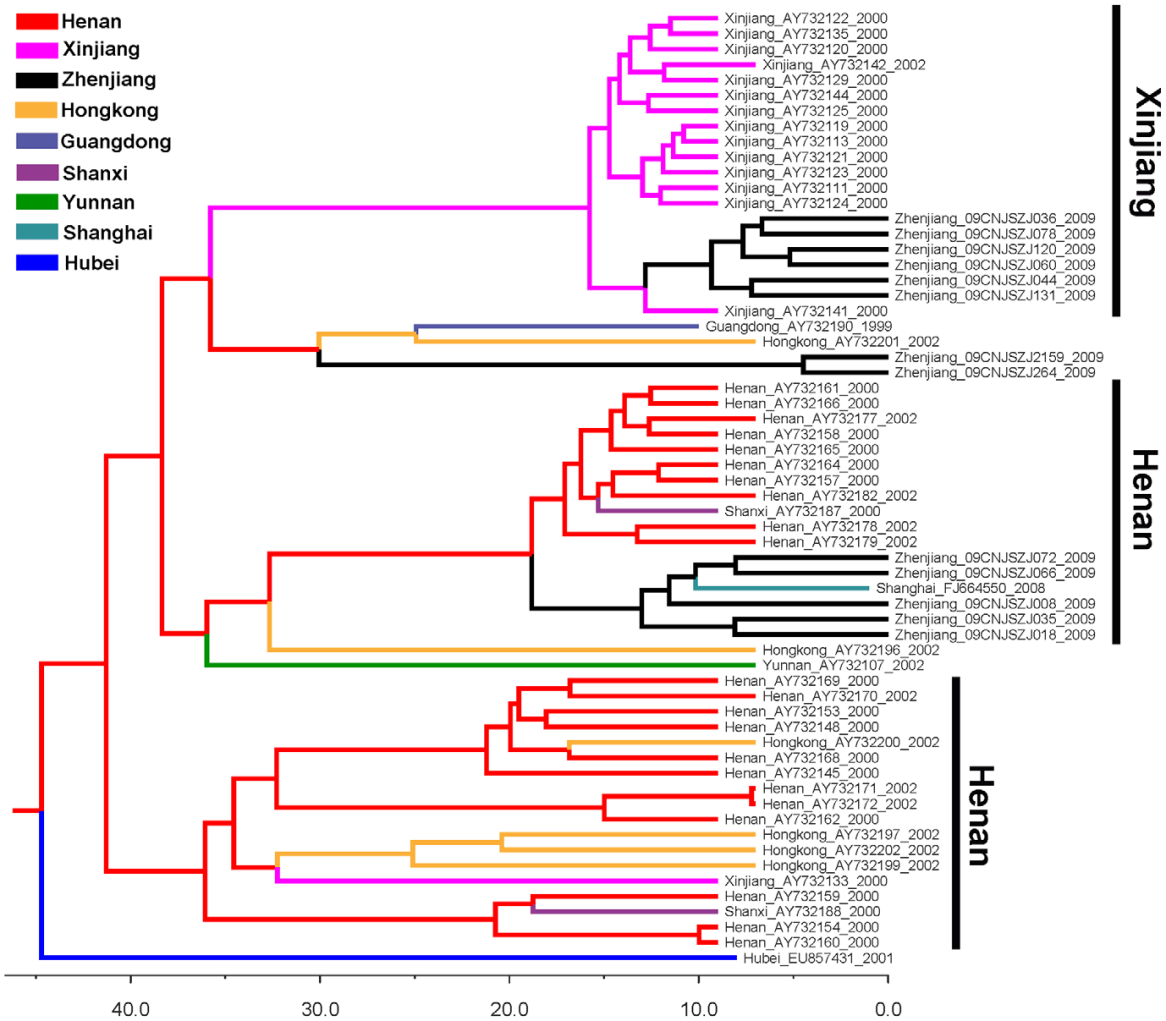
Maximum clade credibility tree of the HCV 1b sequences. The tree was reconstructed based on the genomic region (H77 1341–1835 nt) located in C/E2. Ancestral geographic states were reconstructed using Bayesian phylogeographic inference framework implemented in the BEAST v1.5.4 package. The tree branches are colored according to their respective geographical locations. The time scale is shown at the bottom.

To further investigate the evolutionary origins of HCV 1b and 3a strains circulating in Zhenjiang, we performed phylogeographic analyses using recently developed Bayesian phylogeographic inference framework. The Maximum clade credibility (MCC) tree of HCV 1b strains shows that the most recent common ancestor (tMRCA) of China 1b strains originates in Henan, a central province of China, and except one strain from Hubei province all China 1b strains form four clades (Fig. 4). The strains from Zhenjiang form three sub-clusters and are dispersed within three clades, clearly indicating that Zhenjiang 1b strains have three independent origins. Among three Zhenjiang sub-clusters, two originate from Henan and another originates from Xinjiang. Intriguingly, one strain from Shanghai clusters within one Henan-originated Zhenjiang sub-cluster (Fig. 4), indicating a spread of HCV 1b strains from Zhenjiang to Shanghai. This suggests that Zhenjiang may serve as a middle station during the spread of HCV 1b strains from Henan to Shanghai.

**Figure 5:**
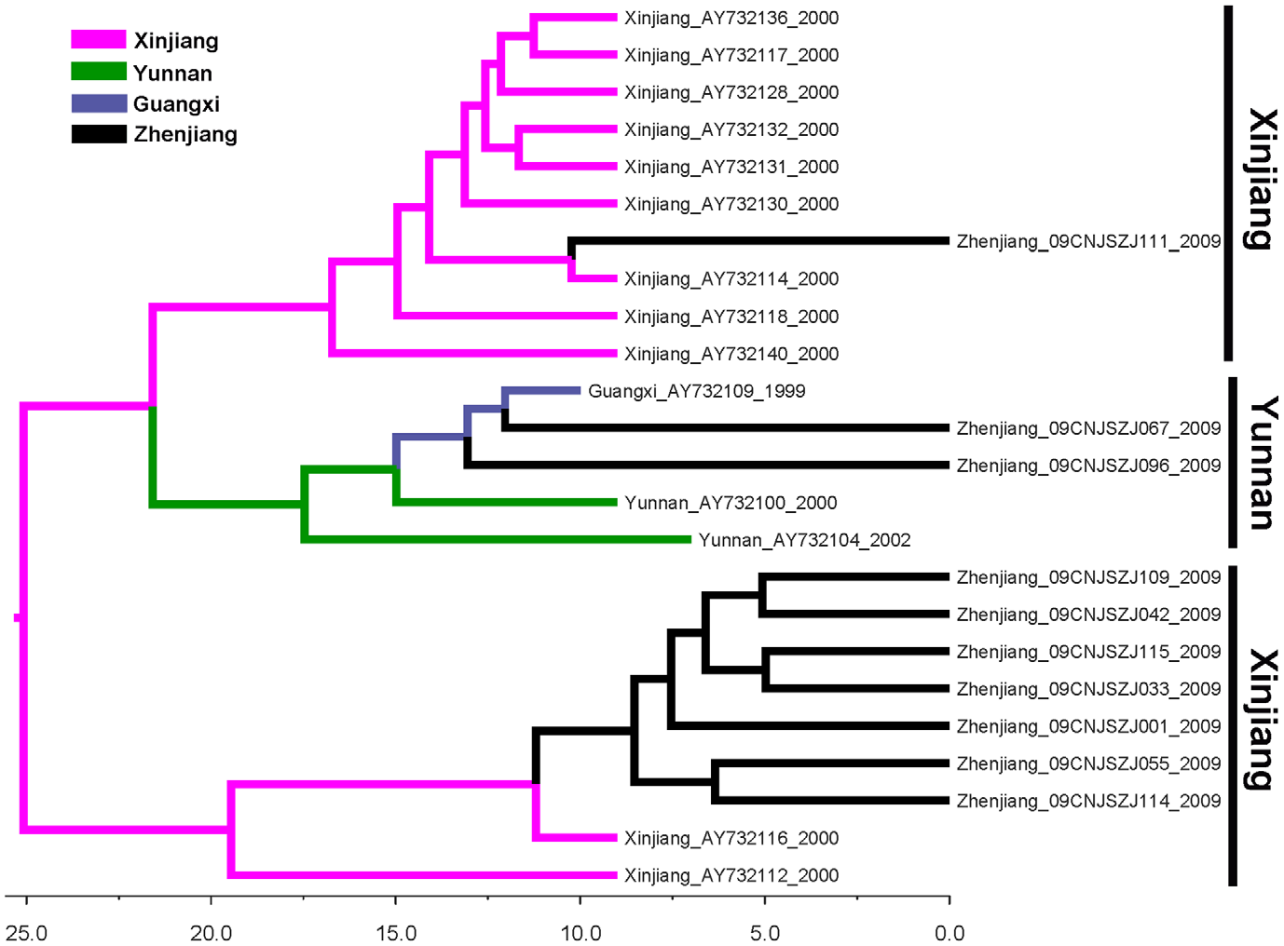
Maximum clade credibility tree of the HCV 3a sequences. The tree was reconstructed based on the genomic region (H77 1356–1826 nt) located in C/E2. Please see Figure 4 for other details.

The tree of HCV 3a strains shows that all 3a strains form three clades with a common origin in Xinjiang (Fig. 5). They include two Xinjiang clades and one Yunnan clade (including strains from Yunnan and Guangxi). Similar to subtype 1b, 3a strains from Zhenjiang are also dispersed within three clades, indicating three independent origins. Two Zhenjiang sub-clusters (one of them only included one sequence) closely cluster within the Xinjiang clades, indicating origins from Xinjiang, and another clusters within the Yunnan clade, implying an origin from Southwestern China.

## Discussion

Currently, 4 HCV genotypes (1, 2, 3 and 6) including more than 13 subtypes are prevalent in China, and the two most predominant subtypes are 1b and 2a, both which result in a nation-wide prevalence [2], [25]. HCV prevalence has been demonstrated to be segregation of subtypes between geographic groups [2], [3], [14]. Among IDUs, the northwestern frontier province Xinjiang and southwestern frontier province Yunnan have the most complex HCV subtype distributions of more than 4 subtypes [8], [10], [20], [21], [25].

In this study, we found that seven HCV subtypes including 1b, 2a, 3a, 3b, 6a, 6e and 6n are circulating among IDUs in Zhenjiang (Table 1). The complex HCV subtype distribution appearing among IDUs in Zhenjiang implies the importance of Zhenjiang in HCV transmission and a complex origination of Zhenjiang strains. Comparison with other regions shows that Zhenjiang has a unique HCV subtype distribution pattern, in which subtypes 3a, 1b and 3b are the three top predominant subtypes, similar to those observed in Xinjiang and Yunnan provinces (Fig. 2). This suggests a close association of Zhenjiang with Xinjiang and Yunnan in HCV transmission, which is supported by further phylogenetic analyses.

Based on the phylogenetic relationship, we investigated the origin of HCV strains circulating among Zhenjiang IDUs. The 3b and 6n strains from Zhenjiang closely cluster with the strains from Yunnan, and Zhenjiang 6e strains closely cluster with the strains from Guangxi (Figs. 3A and S2A), suggesting that these strains are transmitted to Zhenjiang from Southwestern China (Yunnan and/or Guangxi). The 6a strains from Zhenjiang are dispersed among the strains from Guangdong and Hong kong, indicating an origination from Southeastern China. The subtype 2a clade is a monophylogenetic clade, in which one strain from Guangdong is located at the outermost phylogenetic branch. This suggests an initial origin of Zhenjiang 2a strains from Guangdong. Intriguingly, the 2a clade is divided by the Zhenjiang strain 09CNJSZJ249 into two geographic groups, the Southern China group and the Central and North China group (Fig. 3A). This supports a transmission route of HCV along the coastal cities from Southern China to Central and North China via Zhenjiang [13].

The two most prevalent subtypes among Zhenjiang IDUs are 3a and 1b. Both subtypes are also predominant in Xinjiang and Yunnan (Fig. 2) [14], [25]. Because 1b is the nationwide prevalent subtype in China, high proportion of 1b prevalence among Zhenjiang IDUs is unsurprising. In the phylogenetic trees, the 1b strains isolated in Zhenjiang are dispersed together with the strains from Northwestern, Northeastern, Northern, Central, Eastern, Southeastern and Southwestern China (Figs. 3B and S2B), suggesting the genetically association of Zhenjiang strains with the strains from other regions. Further phylogeographic analysis shows that subtype 1b strains are transmitted to Zhenjiang from Henan and Xinjiang via three independent transmission routes and from Zhenjiang one Henan-originated strain is further transmitted eastward to Shanghai (Fig. 4). These results suggest that Zhenjiang might be a middle station in transmission of HCV subtype 1b in China. For 3a subtype, two obvious sub-clusters show that Zhenjiang strains are related to two sub-groups from Xinjiang and Yunnan (Figs. 3C and S2C). Further phylogeographic analysis shows that 3a strains in Zhenjiang are transmitted from Xinjiang and Yunnan (Fig. 5).

**Figure 6:**
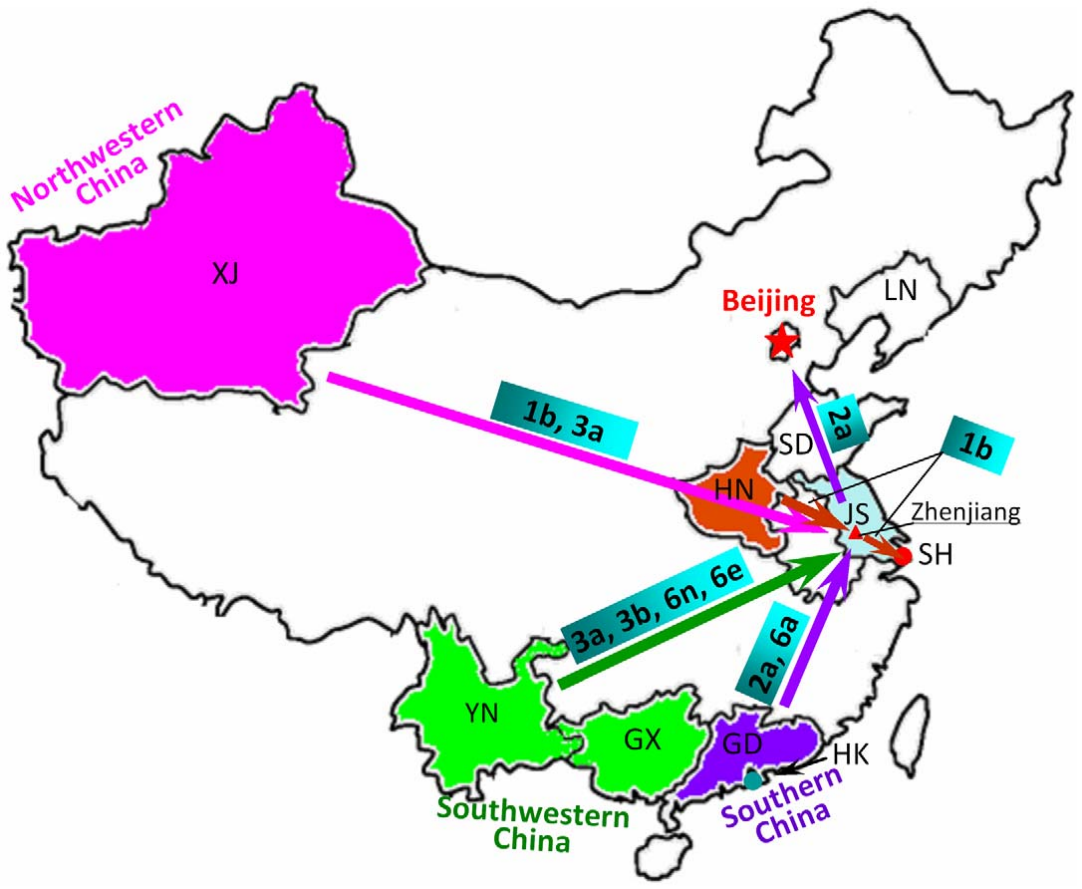
Potential migration routes of several HCV subtypes in China. The arrows represent the potential migration routes of HCV. XJ: Xinjiang; LN: Liaoning; SD: Shandong; HN: Henan; JS: Jiangsu; SH: Shanghai; YN: Yunnan; GX: Guangxi; GD: Guangdong; HK: Hong Kong. Because few corresponding sequences from Taiwan are available for phylogenetic analyses, this Figure does not include Taiwan.

In order to well show the association of Zhenjiang in HCV transmission with other regions of China, we summarize our results in Figure 6. Among seven HCV subtypes circulating among Zhenjiang IDUs, subtypes 3b, 6n and 6e originate from Southwestern China (i.e. Yunnan and/or Guangxi), subtypes 2a and 6a from Southern China (i.e. Guangdong and/or Hong Kong), subtype 1b from Central (i.e. Henan) and Northwestern (i.e. Xinjiang) China, and subtype 3a from Southwestern (i.e. Yunnan) and Northwestern (i.e. Xinjiang) China. From Zhenjiang, subtypes 1b and 2a further spread to Eastern (i.e. Shanghai) and Northern (e.g. Beijing and Liaoning) China, respectively (Fig. 6). These suggest that Zhenjiang may play a crucial role in HCV transmission possible by serving as a middle station. Because Zhenjiang is an important transportation node connecting other regions of China and Eastern China (e.g. Shanghai), the mixing of seven HCV subtypes in Zhenjiang may be associated with large-scale population migration. In addition, we believe that other regional central cities (e.g. Nanjing, Zhengzhou, etc.) in the transportation networks should also play crucial roles in HCV transmission in China.

On the other hand, four HCV subtypes (3a, 3b, 6n and 6e) are demonstrated to spread to Zhenjiang from Yunnan, and another two subtypes (1b and 3a) from Xinjiang (Figs. 4 and 5), suggesting that both Yunnan and Xinjiang play important roles in HCV transmission. In fact, Yunnan that borders the heroin-producing areas (Golden Triangle) is the most important transfer station for drug-trafficking from ‘Golden Triangle’ to other regions of China [26], [27]. Its importance in HIV-1 transmission is well known. Along the drug-trafficking route from Golden Triangle to Yunnan, HIV-1 subtypes B' and C are demonstrated to be introduced into Yunnan from Thailand and India, respectively [26], [27]. The co-circulation of B' and C among IDUs leads to the generation of two closely related CRFs (07_BC and 08_BC), both of which further spread to other regions of China along different drug-trafficking routes [28], [29]. Xinjiang borders another drug-producing area (Golden Crescent) and is another most important transfer station for drug-trafficking to China (Fig. 6) [30]. Up to now, however, there is less report about the role that Xinjiang plays in HIV-1 transmission in China. HCV has similar transmission routes to HIV. Our finding suggests that unlike previously thought, Xinjiang may play an important role in HCV transmission, as well as HIV transmission.

Since the sample size of Zhenjiang IDUs is relatively small and the related data of other regions of China in PubMed is limited, this study is unable to provide the most comprehensive scenario about HCV transmission in China. To deeply understand HCV transmission/migration pattern among IDUs in China, large-scale molecular epidemiological investigations need to be conducted among IDUs population.

## Materials and Methods

### Ethics Statement

This study was done according to the Helsinki II Declaration and was approved by the medical ethics committee of Zhenjiang Center of Disease Control and Prevention (CDC). All participants were voluntary in this study and written informed consents were obtained from them.

### Samples, HCV RNA extraction, RT-PCR amplification

A total of 141 whole blood samples (about 5 mL) were collected from approximate 630 IDUs enrolled in Zhenjiang Methadone Treatment Center and Jiangsu Women's Re-education through Labor Camp using sterile ethylenediaminetetraacetic acid tubes during 2009. Both institutions are located in Zhenjiang city of Jiangsu province and all participants are local residents. The information about the gender and the age of these participants in this study was not collected. The samples were transported from the scene to Zhenjiang CDC for detection of antibodies to HCV. However, because of neglect of official duty in preservation and/or transportation of samples, some samples were exposed at room temperature two days before store at −80°C, which may result in a low amplification efficiency of viral genome. Plasma was separated by centrifugation and anti-HCV antibodies were determined using an enzyme immunoassay kit (Wantai Biotechnology Ltd., Beijing, China). The RNA was extracted from 200 µl of HCV-positive plasma with the MiniBEST Viral RNA/DNA Extraction Kit Ver 4.0 (TaKaRa Biotechnology Co. Ltd., Dalian, China). The cDNA was synthesized using the TaKaRa RNA PCR kit (AMV) Ver.3.0 and then was subjected to the nested PCR for amplifying the C/E2 and the NS5B regions of HCV genome. The first PCR reactions were performed using the same kit as the cDNA synthesis, and the second PCR reactions were performed using TaKaRa PCR Amplification Kit (TaKaRa, Dalian, China). The primer pairs used in the nested PCR were modified from the previous paper. The information of primers and the reaction programs are shown in Table S1. After the identification using 1% agarose gel, the amplified products were sent to Shanghai Invitrogen Biotechnology Co., LTD. for sequencing.

### HCV genotyping and phylogenetic analyses

The sequences obtained here were aligned together with HCV subtype reference sequences using CLUSTAL W program implemented in MEGA 4.0. HCV subtype reference sequences were downloaded from the Los Alamos HCV database (http://hcv.lanl.gov/content/sequence/HCV/ToolsOutline.html). HCV sequences from other regions of China were obtained from refs [8], [9], [10], [20], [21], [22], [23], [24]. The phylogenetic trees of two HCV genomic fragments NS5B and C/E2 were constructed using NJ (neighbor-joining) method (MEGA 4.0) [31], and the reliability of the trees were evaluated by the bootstrap method with 1,000 replications.

### Phylogeographic analyses of HCV 1b and 3a

To investigate the possible geographic origins of HCV 1b and 3a in Zhenjiang, all HCV 1b and HCV 3a nucleotide sequences with known sampling years and locations in China were collected from the HCV Sequence Database. Each sequence was assigned a character state reflecting its sampling location. The maximum clade credibility tree was constructed using a MCMC (Markov Chain Monte Carlo) method implemented in the BEAST v1.5.4 package [32]. Ancestral geographic states were inferred using a geographically explicit Bayesian MCMC method implemented in BEAST v1.5.4 package [32], [33]. MCMC analysis was run for 20 million generations, with sampling every 10,000 generations. The initial 25% of the samples were discarded as burn-in, leaving 1501 trees per run, when we summarized the trees using TreeAnnotator implemented in the BEAST v1.5.4 package. Because the corresponding sequences from other China regions are very limited, we did not perform the phylogeographic analyses of other HCV subtypes.

### Nucleotide sequence accession numbers

The HCV NS5B and C/E2 sequences reported in this article are available in GenBank under accession numbers of HQ318826-HQ318927.

## Supporting Information

Figure S1 **Phylogenetic tree of C/E2 fragments of HCV strains isolated from Zhenjiang.** For other details, please see Figure 1.
(DOC)

Figure S2 **The C/E2 phylogenetic relationship of HCV strains isolated from Zhenjiang IDUs with those from other regions of China.** **A,** whole tree; **B,** Subtype 1b subtree; **C,** Subtype 3a subtree. For other details, please see Figure 3.
(DOC)

Table S1 **Information of the primer pairs used in this study.**
(DOC)
