# Supplementary material for: HCV Subtype Characterization among Injection Drug Users: Implication for a Crucial Role of Zhenjiang in HCV Transmission in China
Source: PLoS One. 2011 Feb 3;6(2):e16817. doi: 10.1371/journal.pone.0016817 (PMC3033423; doi:10.1371/journal.pone.0016817)
Supplement: Figure S1 — Phylogenetic tree of C/E2 fragments of HCV strains isolated from Zhenjiang. For other details, please see Figure 1. (DOC) [file pone.0016817.s001.doc]

**Figure S1. Phylogenetic tree of C/E2 fragments of HCV strains isolated from Zhenjiang.** For other details, please see Figure 1.
