# Supplementary material for: HCV Subtype Characterization among Injection Drug Users: Implication for a Crucial Role of Zhenjiang in HCV Transmission in China
Source: PLoS One. 2011 Feb 3;6(2):e16817. doi: 10.1371/journal.pone.0016817 (PMC3033423; doi:10.1371/journal.pone.0016817)
Supplement: Figure S2 — The C/E2 phylogenetic relationship of HCV strains isolated from Zhenjiang IDUs with those from other regions of China. A, whole tree; B, Subtype 1b subtree; C, Subtype 3a subtree. For other details, please see Figure 3. (DOC) [file pone.0016817.s002.doc]

**Figure S2. The C/E2 phylogenetic relationship of HCV strains isolated from Zhenjiang IDUs with those from other regions of China. A,** whole tree; **B,** Subtype 1b subtree; C**,** Subtype 3a subtree. For other details, please see Figure 3.
