# Supplementary material for: HCV Subtype Characterization among Injection Drug Users: Implication for a Crucial Role of Zhenjiang in HCV Transmission in China
Source: PLoS One. 2011 Feb 3;6(2):e16817. doi: 10.1371/journal.pone.0016817 (PMC3033423; doi:10.1371/journal.pone.0016817)
Supplement: Table S1 — Information of the primer pairs used in this study. (DOC) [file pone.0016817.s003.doc]

**Table S1. Information of the primer pairs used in this study.**

| **Region** | **Nested PCR** | **Name** | **Sequences（5’-3’）** | **Position in H77 genome** | **Size (bp)** | **Reaction program** |
| --- | --- | --- | --- | --- | --- | --- |
| **C/E2** | 1^st^ PCR | C/E2-F1 | GCCGACCTCATGGGGTACAT | 732-751 | 1480 | 94℃ for 2 min, 35 cycles of 94℃ for 30 sec, 50℃ for 30 sec and 72℃ for 1.5 min, with final extension at 72℃ for 10 min |
|  |  | C/E2-R1 | ARTTBTYDGTRCANGGRTARTGCCA | 2187-2211 |  |  |
|  | 2^nd^ PCR | C/E2-F2 | CCYGGTTGCTCYTTYTCTATCTT | 849-871 | 1303 | 94℃ for 2 min, 35 cycles of 94℃ for 30 sec, 55℃ for 30 sec and 72℃ for 1.5 min, with final extension at 72℃ for 10 min |
|  |  | C/E2-R2 | GTNADCCANGGNCCNGMNCCRCA | 2130-2152 |  |  |
| **NS5B** | 1^st^ PCR | NS5B-F1 | GGSTTYTCNTATGAYACCMGVTGYTTTGA | 8247-8275 | 1104 | 94℃ for 2 min, 35 cycles of 94℃ for 30 sec, 50℃ for 30 sec and 72℃ for 1.5 min, with final extension at 72℃ for 10 min |
|  |  | NS5B-R1 | CTACCCCTACNGHDAGTAGGAGTAGGC | 9325-9351 |  |  |
|  | 2^nd^ PCR | NS5B-F2 | GCTGYTTTGAYTCAACNGTCAC | 8266-8287 | 1036 | 94℃ for 2 min, 35 cycles of 94℃ for 30 sec, 55℃ for 30 sec and 72℃ for 1.5 min, with final extension at 72℃ for 10 min |
|  |  | NS5B-R2 | GRGCHYGVGACACGCTGTGATANATGTC | 9276-9303 |  |  |
